# Identification of housekeeping genes of *Candidatus* Branchiomonas cysticola associated with epitheliocystis in Atlantic salmon (*Salmo salar* L.)

**DOI:** 10.1007/s00203-022-02966-y

**Published:** 2022-06-04

**Authors:** Even Bysveen Mjølnerød, Aashish Srivastava, Lindsey J. Moore, Heidrun Plarre, Are Nylund

**Affiliations:** 1 Department of Biological Sciences, Fish Disease Research Group, University of Bergen, PO Box 7803, 5020 Bergen, Norway; 2 Department of Clinical Science, University of Bergen, PO Box 7804, 5020 Bergen, Norway

**Keywords:** *Candidatus* Branchiomonas cysticola, Epitheliocystis, In-situ hybridization, Next-generation sequencing

## Abstract

*Candidatus* Branchiomonas cysticola is an intracellular, gram-negative *Betaproteobacteria* causing epitheliocystis in Atlantic Salmon (*Salmo salar* L.). The bacterium has not been genetically characterized at the intraspecific level despite its high prevalence among salmon suffering from gill disease in Norwegian aquaculture. DNA from gill samples of Atlantic salmon PCR positive for *Cand.* B. cysticola and displaying pathological signs of gill disease, was, therefore, extracted and subject to next-generation sequencing (mNGS). Partial sequences of four housekeeping (HK) genes (*aceE, lepA, rplB, rpoC*) were ultimately identified from the sequenced material. Assays for real-time RT-PCR and fluorescence *in-situ* hybridization, targeting the newly acquired genes, were simultaneously applied with existing assays targeting the previously characterized 16S rRNA gene. Agreement in both expression and specificity between these putative HK genes and the 16S gene was observed in all instances, indicating that the partial sequences of these HK genes originate from *Cand.* B. cysticola. The knowledge generated from the present study constitutes a major prerequisite for the future design of novel genotyping schemes for this bacterium.

## Introduction

Gill diseases (GD) represent an ever-increasing health related as well as purely economic issue in Norwegian aquaculture. It is a multifactorial disease that contributes to considerable mortality of farmed Atlantic salmon (*Salmo salar*) annually in Norway (Kvellestad et al. 2005; Steinum et al. 2009, 2010a; Nylund et al. 2011). Epitheliocystis represents one of the most prevalent conditions associated with GD in farmed Atlantic salmon (Nylund et al. 1998, 2015; Draghi et al. 2004; Gjessing et al. 2021) and has been reported from over 90 different fish species worldwide (Bradley et al. 1988; Meijer et al. 2006; Nowak and LaPatra 2006; Horn 2008; Karlsen et al. 2008; Polkinghorne et al. 2010; Schmidt-Posthaus et al. 2012; Steigen et al. 2013; Fehr et al. 2013; Stride and Nowak 2014). Epitheliocystis in farmed salmon is characterized by intracytoplasmic, intravacuolar, inclusions containing gram-negative bacteria belonging to Chlamydiales, primarily found in epithelial cells lining the secondary lamella of the gills (Nylund et al. 1998) (Draghi et al. 2004; Nowak and LaPatra 2006; Horn 2008; Karlsen et al. 2008; Gunnarsson et al. 2017). One exception is the betaproteobacterium *Candidatus* Branchiomonas cysticola, that also causes epitheliocystis in salmon (Toenshoff et al. 2012; Mitchell et al. 2013). Following its first description, *Cand.* B. cysticola has since been described as the most prevalent bacterial agent in salmon displaying pathological signs of epitheliocystis (Steinum et al. 2010b; Toenshoff et al. 2012; Mitchell et al. 2013).

*Cand.* B. cysticola has yet to be genetically characterized beyond the 16S rRNA gene (Toenshoff et al. 2012; Mitchell et al. 2013). This is mainly due to the obligate intracellular nature of the bacterium and the lack of in vitro/in vivo cultivation systems. The latter complicates the development of genotyping schemes for this bacterial agent. Sequencing of novel microorganisms have during recent years been heavily dependent on homogenous cultures of the respective agents (Lasken and McLean 2014). One exception is the epitheliocystis agent *Candidatus* Syngnamydia salmonis that can be cultured in *Paramoeba perurans* which made it possible to obtain a large part of the genome of this member of Chlamydiales (Nylund et al. 2018b). Culture-independent methods such as next-generation sequencing (mNGS) have thus been increasingly applied to uncover the full and/or partial genomes of novel pathogenic microorganisms associated with diseases in fish (Tengs and Rimstad 2017; Skoge et al. 2018; Sandlund et al. 2021).

In the present study, identification of housekeeping (HK) genes from *Cand.* B. cysticola was conducted using material for next generation sequencing (NGS) technology. The unculturable nature of this bacterium also required in situ hybridization for visualization of the spatial expression for the newly acquired genes, confirming their association with the epitheliocysts. Fluorescence in situ hybridization (FISH) on fixed gill tissue was, therefore, performed by simultaneous application of a *Cand.* B. cysticola-specific oligonucleotide probe (Toenshoff et al. 2012) and newly designed probes targeting putative HK genes.

The characterization of novel *Cand.* B. cysticola-specific sequences enables the development of genotyping schemes for phylogenetic reconstruction of the bacterium’s population structure. A multilocus phylogenetic analysis (MLPA) scheme, comprising newly identified genes, could provide valuable knowledge of epidemiologic relevance for the prevention of gill diseases in Norwegian aquaculture. Elucidation of the bacterium’s population structure represents a major prerequisite to acquire new diagnostic tools, identify strains of varying virulence, and to develop possible future vaccines. In the present study we established a proof of concept for sequencing and identification of *Cand.* B. cysticola HK genes through mNGS and FISH, enabling the future phylogenetic characterization of this bacterium.

## Materials and methods

### Sample processing and analysis

Lethargic Atlantic salmon (*Salmo salar*) displaying increased ventilation rate, pale and mottled gills were sampled by collecting the left-side second gill arch. Each gill sample was preserved frozen on dry ice for subsequent DNA/RNA extraction and in 4% buffered formaldehyde solution for histological analysis. DNA was extracted from *Cand.* B. cysticola-positive gills using the E.Z.N.A^®^ Tissue DNA Kit (Omega Bio-Tek, Norcross, USA) in accordance with the manufacturers protocol. RNA was extracted using the protocol for Isol-RNA Lysis Reagent as described by Gunnarsson et al. (2017). Relative quantification of *Cand*. B. cysticola and associated gill pathogens (*Cand.* Piscichlamydia salmonis, *Cand.* Syngnamydia salmonis, *Paramoeba perurans*) present in the gill tissues were performed using real-time RT-PCR (Table 1). Two samples (g4 and g42) were selected based on this relative quantification. The eukaryotic elongation factor 1 alpha (EF1A_A_, Table 1) from salmon (Olsvik et al. 2005) was used as a control of the RNA amount, and a good extraction was expected to give a Ct-value around 15 (the cycle threshold was set to 0.1).

**Table 1:** Real-time RT-PCR assays used in the study

Assay	Gene target	Primer and probe	Sequences (5'–3')	Amplicon size	Reference
aceE	Pyruvat dehydrogenase E1 component	F primer	CTGGAGGCGTCTCTTTATGGA	60 bp	This study
		Probe	FAM-AGCAGCCGCTACCA-MGB		
		R primer	TGGGTAAATGGTGGACGCTATA		
lepA	Elongation factor 4 (EF4)	F primer	GCGGAAATTGAAGATATGATTGG	64 bp	This study
		Probe	FAM-TTGATGCCAGTCGAGC-MGB		
		R primer	CGGTTTTCGCACTACAAGGAA		
rplB	50S ribosomal protein L2	F primer	CACCACGATGCCTGACTGTAA	59 bp	This study
		Probe	FAM-ACCATGCACATTACG-MGB		
		R primer	CAAGCGTGCGGCTGTTG		
rpoC	DNA-directed RNA polymerase subunit β	F primer	CGTCGGGTCAAATATCCTGAA	64 bp	This study
		Probe	FAM-TCGCTGTTTTTAACGCTG-MGB		
		R primer	GGGCACGAAAAGGGTTAGC		
EF1A_A_	Eukaryotic elongation factor 1A	F primer	CCCCTCCAGGACGTTTACAAA	57 bp	(Olsvik et al. 2005)
		Probe	FAM-ATCGGTGGTATTGGAAC-MGB		
		R primer	CACACGGCCCACAGGTACA		
Epit	*Cand.* B. cysticola 16S rRNA	F primer	GAGTAATACATCGGAACGTGTCTAGTG	84 bp	(Nylund et al. 2018a)
		Probe	FAM-ACTTAGCGAAAGTTAAGC-MGB		
		R primer	CTTTCCTCTCCCAAGCTTATGC		
Pperu	*Paramoeba perurans* 18S rRNA	F primer	GATAACCGTGGTAAATCTAGAGCTAATA	101 bp	(Nylund et al. 2018b)
		Probe	FAM-CTGGTTCTTTCGRGAGC-MGB		
		R primer	TGGCATTGGCTTTTGAATCT		
SCh	*Cand.* S. salmonis 16S rRNA	F primer	GGGTAGCCCGATATCTTCAAAGT	66 bp	(Nylund et al. 2015)
		Probe	FAM-TCCTTCGGGACCTTAC-MGB		
		R primer	CCCATGAGCCGCTCTCTCT		
PCh	*Cand.* P. salmonis 16S rRNA	F primer	TCACCCCCAGGCTGCTT	60 bp	(Nylund et al. 2008)
		Probe	FAM-CAAAACTGCTAGACTAGAGT-MGB		
		R primer	GAATTCCATTTCCCCCTCTTG		

* F* forward,* R* reverse,* FAM* 6-carboxyfluorecein,* MGB* minor groove binder

The integrity of total DNA extracted was assessed by 1% agarose gel electrophoresis. High molecular weight DNA (> 5 Kb) was subsequently extracted from the gel of sample g4 and further purified using the E.Z.N.A^®^ Gel Extraction Kit according to the manufacturers protocol. Total DNA irrespective of molecular weight was preserved from sample g42 for downstream application. Total DNA quantities were assessed by UV–Vis Spectrometry using the Nanodrop^®^ ND-1000 (Thermo Fisher, Waltham, USA), while relative quantification of target and host DNA was performed using real-time PCR (Table 1).

### NGS sequencing and gene mining

Two samples, each containing five micrograms of purified DNA were sent to the Genomics Core Facility (GCF) at Haukeland University Hospital (Bergen, Norway) for shotgun sequencing. The NGS library was prepared using the Nextera XT DNA Library Preparation Kit (Illumina, San Diego, USA) with a target insert size of 300 bp paired end reads. The final prepped samples were sequenced through a 600-cycle flow cell using the Illumina NextSeq 4000.

Cutadapt v3.4 (Martin 2011) was used to remove sequencing adapters and bases below a Phred quality score of 30. To remove the host contamination the cleaned reads were aligned to *Salmon salar* whole genome (GCF_000233375.1) downloaded from NCBI GenBank using bowtie2 v2.2.9. (Langmead and Salzberg 2012). The aligned reads were removed, while unaligned reads were further cleaned using Trimmomatic v0.39. (Bolger et al. 2014). De-novo assembly of trimmed sequence reads was performed using SPAdes genome assembly algorithm (Bankevich et al. 2012). Quality of the obtained assembly was assessed with Quast v5.0.2 (Mikheenko et al. 2018). This whole genome shotgun project has been deposited at DDBJ/ENA/GenBank under the accession JAKNSC000000000. The version described in this paper is version JAKNSC010000000.

Finally assembled scaffold sequences were imported into Geneious Prime (2021), where a Basic Local Alignment Search Tool (BLAST) database was generated comprising all contigs assembled. The annotated genome of *Taylorella aquigenitalis* (Betaproteobacteria; Burkholderiales; Alcaligenaceae; Taylorella) was imported from NCBI GenBank (NZ_CP021060.1) as a putative reference genome and subsequently aligned against the local BLAST database. Four homologous sequences that aligned with annotated housekeeping (HK) genes of *T. aquigenitalis* (*aceE, lepA, rplB* and *rpoC*) were extracted and subject to primer/probe design for real-time RT-PCR (Table 1) and FISH (Table 2). Real-time RT-PCR was performed on RNA from previously obtained gill samples proven positive (*n* = 5) and negative (*n* = 5) for *Cand.* B. cysticola, for determining the efficiency and specificity of the assays.

**Table 2:** FISH probes

Probe	Sequence (5´–3´)	Fluorophore (5´End, 3´End)	Reference
aceE	TAGGCACAGAAGCAGCCATC	Cyanine 3	This study
lepA	TTCAATTTCCGCACGCACTG	Cyanine 3	This study
rplB	TTGTGCATACGACCCCTCAC	Cyanine 3	This study
rpoC	TGCCCCATGGGTAGAGATGA	Cyanine 3	This study
BraCy-129	CCCACCACTAGACACGTT	Cyanine 5	(Toenshoff et al. 2012)

Fluorescently labelled oligonucleotide probes targeting *Cand*. B. cysticola HK genes (Cy3) identified by mNGS and *Cand*. B. cysticola specific oligo probe BraCy-129 (Cy5) (Toenshoff et al. 2012*) *targeting 16S rRNA

### Fluorescence in-situ hybridization (FISH)

#### Pretreatment of gill sections

Fixed gill arches were paraffin embedded by Pharmaq Analytiq (Bergen, Norway). Formalin-fixed paraffin embedded (FFPE) gill samples were sectioned at 5 μm thick using a RNAse treated microtome and diethylpyrocarbonate (DEPC) water for processing. The sections were deposited onto SuperFrost^®^ Plus slides (Thermo Fisher, Waltham, USA) and dried at 58 ℃ for 20 min before being deparaffinized by three baths of xylene for 5 min. The sections were rehydrated through successive baths of declining ethanol concentrations in DEPC water (2 × 100%, 95%, 70% and 50% ethanol) for 1 min each. Irreversible inactivation of endogenous RNAses was achieved by exposing the sections to 0.1% of active DEPC in PBS for 12 min at room temperature (RT) (Pernthaler and Amann 2004). The slides were subsequently immersed in 2 × saline sodium citrate buffer solution (SSC) for 2 × 1 min. Protein digestion enabling intracellular/intravacuolar probe penetration was performed by incubating the slides in 0.7 μg proteinase K (2 mg/mL) (from *Tritirachium album*; ≥ 30 units/mg protein; Merck, Darmstadt, Germany) in 0.1 M Tris–HCl and 50 mM EDTA (pH8) for 15 min at 37 ℃ (Toenshoff et al. 2012). Slides were washed in 2 × SSC for 2 × 5 min and dehydrated in increasing ethanol concentrations in DEPC water (50%, 70%, 95% and 2 × 100% EtOH) for 1 min each. The slides were finally air dried for ≥ 1 h at RT before the sections were circled using a hydrophobic marker pen.

### FISH of mRNA

1 mL of hybridization buffer; 900 mM NaCl, 20 mM Tris–HCl pH8, 35% deionized formamide, 0.01% SDS in DEPC, was prepared in a RNAse free 1.5 mL microtube. Aliquots of 180 μl hybridization buffer were made for each section. 2 μl of oligo probes labeled with Cyanine 3 (Cy3) targeting the individual housekeeping genes (40 ng/μl) were added to separate aliquots of hybridization buffer together with 2 μl of oligo probe BraCy-129 (40 ng/μl) labeled with Cyanine 5 (Cy5) (Table 2). The final volume of hybridization solution containing the two probes was then deposited onto separate tissue sections. The slides were placed within an airtight humidity chamber containing Whatman paper soaked in 2 × SSC and incubated at 46 ℃ for 2 h. Following hybridization, the slides were transferred to 50 mL of wash buffer; 20 mM Tris–HCl pH 8, 70 mM NaCl, 50 mM EDTA, 0.01% SDS in Milli-Q water and incubated at 48 ℃. The slides were finally immersed in 4 ℃ Milli-Q water for a couple of seconds before being air-dried at RT with minimal light exposure. Dried slides were mounted in Fluoroshield™ histology mounting medium (Merck, Darmstadt, Germany) and images were captured using the Leica AF6000 fluorescence imaging system. Sequential and simultaneous processing of images was performed using the Leica Application Suite X (version 3.7.4) imaging software.

## Results

**Fig. 1:**
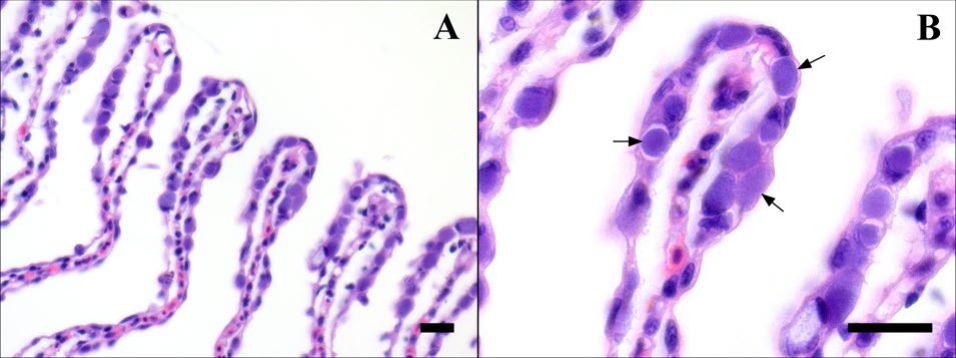
Haematoxylin and eosin (HE) stained FFPE sections of gills from farmed Atlantic salmon in seawater displaying epitheliocystis. (**A**) An abundance of basophilic (deep purple) intracellular inclusions of bacteria within epithelial cells located apically on multiple secondary lamellae (40×) (**B**). Morphology of cysts (arrows) demonstrating the intracellular nature of membrane delimited bacteria occupying the majority of the cytoplasmic volume (100×) Scale bars represents 20 µm

Histological analyses of sampled gills showed common pathological signs of GD including circulatory disturbances, hyperplastic/hypertrophic epithelial cells, inflammation and necrosis, as well as numerous intracellular inclusions of bacteria accordant with epitheliocystis (Fig. 1). Real-time RT-PCR on RNA extracted from samples g4 and g42 targeting *Cand.* B. cysticola 16S rRNA/ salmon EF1A_A_ resulted in Ct-values of 8.9/14.3 and 7.3/12.0, for these two samples, respectively. These values indicate high levels of *Cand*. B. cysticola suggesting that the observed epitheliocysts seen in the gill tissues could be caused by this bacterium.

### NGS and gene mining

Illumina sequencing of extracted DNA produced 2.7 million and 4.2 million paired end reads from samples g4 and g42 following host contaminant removal and sequence clean-up. Assembly of finally processed reads generated 350 and 2803 contigs for these samples, respectively.

NCBI GenBank nucleotide BLAST search (BLASTn) enabled identification of putative *Cand.* B. cysticola specific sequences from the assembled scaffold sequences. A contig of 1541 bps was retrieved (OL314810) demonstrating 99.93% nucleotide identity and 100% query cover with the partial 16S rRNA gene sequence deposited by Toenshoff et al. (2012) (JN968376.1). Alignment of the putative reference genome of *Taylorella equigenitalis* using the custom BLAST database of assembled scaffolds resulted in 68 hits and the extraction of four sequences homologous to annotated HK genes (*aceE, lepA, rplB, rpoC*) of *T. aquigenitalis*.

### Real-time RT-PCR

Real-time RT-PCR on RNA from previously obtained gill samples, generated amplicons for all loci when assays targeting the newly acquired HK genes (Table 1) were applied, showing the presence of these in the *Cand*. B. cysticola-positive RNA. No amplicons were detected among negative samples (Table 3). The results also demonstrated consistent expression levels of all four genes (*aceE, lepA, rplB, rpoC*) though at significantly higher Ct-values compared to the ‘Epit’ assay targeting 16S rRNA (Table 3).

**Table 3:** Real-time RT-PCR results

Assay	Ct value range, positive fish (*n* = 5)	Ct value range, negative fish (*n* = 5)
EFA1a	14–15	16–18
Epit	7–13	Undetected
aceE	19–20	Undetected
lepA	21–23	Undetected
rplB	19–20	Undetected
rpoC	19–21	Undetected

Ct-value range for the selected assays

### Fluorescence in situ hybridization (FISH)

**Fig. 2:**
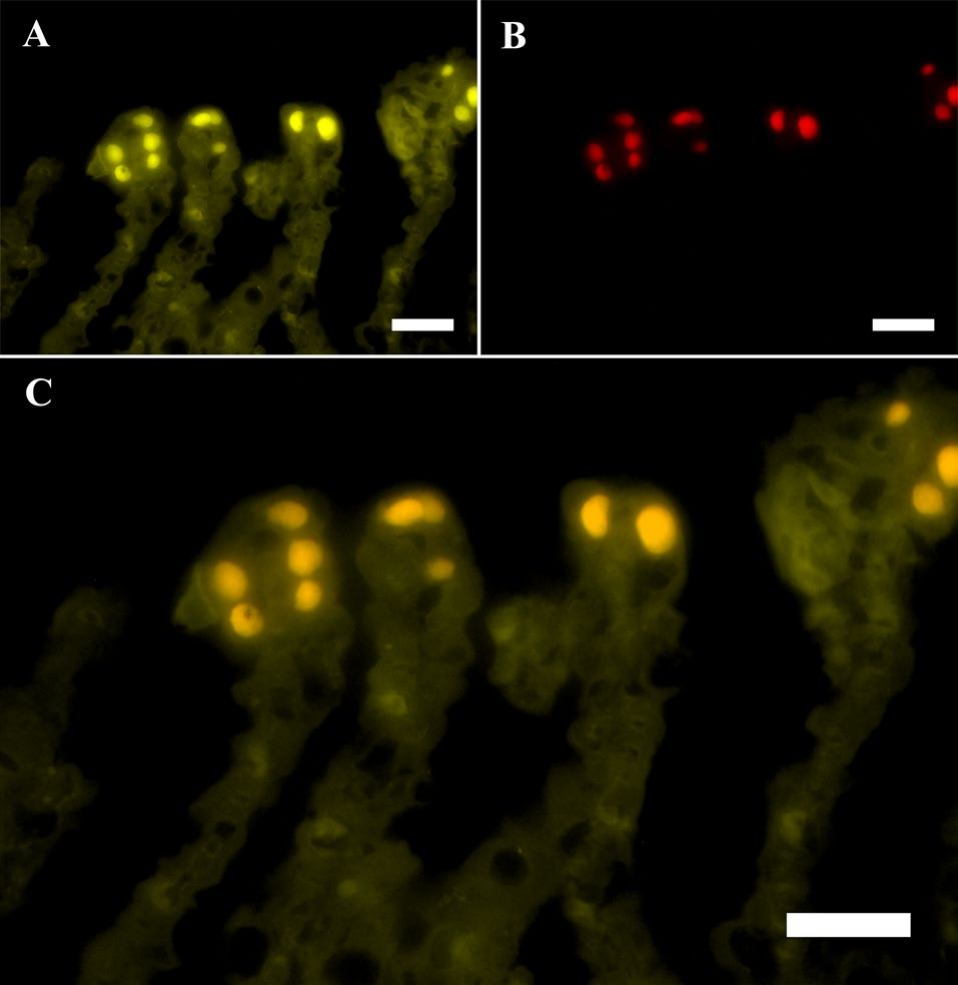
FISH of mRNA. Fluorescently labeled oligonucleotide probes BraCy-129 (Cy5, yellow) targeting *Cand*. B. cysticola 16S rRNA (**A**) and 50S ribosomal protein L2 (*rplB*, Cy3, red) (**B**) from identical intracellular structures located apically in the secondary lamellae of the gills accordant with epitheliocysts. Overlay of the separate fluorophores demonstrates the co-localizing signal (orange) of the two FISH-probes combined (**C**) The faint fluorescent signal visualizing the silhouette of the secondary lamellae (**A, C**) represents auto fluorescence of the gill tissue due to the similar emission spectrum of Cy5. Magnification is 100 × and scale bars represents 20 µm

Simultaneous application of oligonucleotide probe BraCy-129 with aceE, lepA, rplB, or rpoC all produced strong fluorescent signals in dense intracellular inclusions resembling the morphology of epitheliocysts (Fig. 2). Sequential visualization of the separate fluorophores (Cy3/Cy5) showed that the fluorescent signals originated from identical structures of the gill (Fig. 2A, B). The co-localizing hybridization of BraCy-129 and probes targeting putative *Cand.* B. cysticola HK genes are further demonstrated by the combined fluorescent signal from both fluorophores (Fig. 2C). The BraCy-129 (Cy5, yellow) probe produced a higher level of emission compared to the Cy3-labelled probes (red) producing a yellow/orange colour when these images were superimposed (Fig. 2C).

## Discussion

*Candidatus* Branchiomonas cysticola has not been genetically characterized beyond the 16S rRNA gene since its first description associated with epitheliocystis in Atlantic salmon (Toenshoff et al. 2012). The design of a FISH probe (Bracy-129) that was shown to be specific to *Cand.* B. cysticola from a background of co-infective agents (e.g., *Candidatus* Piscichlamydia salmonis) (Toenshoff et al. 2012; Mitchell et al. 2013), has now enabled the identification of several HK *Cand.* B. cysticola genes. Through simultaneous application of ‘BraCy-129’ and FISH probes targeting the loci of putative *Cand.* B. cysticola HK genes acquired by mNGS, we have established a proof of concept for the characterization HK genes specific to this bacterium.

Examining the gills sampled from lethargic salmon displaying clinical signs of GD showed typical histopathological changes associated with epitheliocystis (Fig. 1). When studying the cysts at 100 × magnification using a light microscope, a resemblance in morphology was observed consistent with previous descriptions of epitheliocysts in Atlantic salmon (Fig. 1B)(Nylund et al. 1998, 2015; Draghi et al. 2004; Karlsen et al. 2008; Toenshoff et al. 2012; Mitchell et al. 2013).

Real-time RT-PCR on total RNA isolated from samples g4 and g42 showed an abundance of *Cand.* B. cysticola. Additional RT-PCR analyses screening for closely associated *Chlamydiales* (Draghi et al. 2004; Nylund et al. 2015) as well as *Paramoeba perurans* (Table 1)*,* revealed moderate amounts of *Cand.* S. salmonis, though no detection of *Cand.* P. salmonis. The levels of *Cand.* S. salmonis can, however, appear misleading when designating an etiological agent as it often coincides with the presence of *Paramoeba perurans*. *Cand.* S. salmonis has previously been found to be present in the majority, but not exclusively within this amoeba and it may also be present in epitheliocysts on the salmon gills (Nylund et al. 2015). Due to the moderate/high amounts of *P. perurans* present in the samples subjected to RT-PCR, detection of *Cand.* S. salmonis is partly attributed to the presence of amoeba and is thus not considered to be the primary agent in causing the abundance of cysts observed by histopathology (Fig. 1). Therefore, *Cand.* B. cysticola was considered the most probable etiological agent in the development of epitheliocystis. This is in agreement with previous studies on agent prevalence and pathology from salmon displaying epitheliocystis associated with associated with GD (Toenshoff et al. 2012; Mitchell et al. 2013; Gjessing et al. 2021).

BLASTn searches of final assembled NGS contigs enabled the identification of *Cand.* B. cysticola specific RNA operons (OL314810), as well as putative reference genomes for local BLAST alignments within Geneious Prime. One such genome was that of *Taylorella aquigenitalis* (NZ_CP021060.1) which was chosen for the retrieval of putative HK genes due to its high BLASTn alignment score for contigs displaying similarity with betaproteobacteria of *Burkholderiales.*

RT-PCR on RNA from previously obtained gill samples applying assays of putative HK genes produced amplicons for all four genes (*aceE, lepA, rplB, rpoC*) among samples positive for *Cand.* B. cysticola (Table 3). Considering the lack of detected amplicons within samples proven negative for this bacterium, these results indicated that the assays were specific to *Cand.* B. cysticola. The invariable range in Ct-value of these assays also showed the uniform expression levels expected of most prokaryotic HK genes (Table 3). However, the Ct-values generated by these assays were higher compared to the ‘Epit’ assay targeting the 16S rRNA gene (Table 3). This was probably due to the highly expressed nature of 16S rRNA and the multiple rRNA operons (*rrn*) usually present within the genome of most bacteria (Klappenbach et al. 2001).

Fluorescence *in-situ* hybridization of mRNA using BraCy-129 produced a signal co-localised with structures displaying a similar size, form, and location (Fig. 2A) as the epitheliocysts recognized by histopathology (Fig. 1B). This observable specificity corresponds with previous applications of this probe describing the etiology of *Cand.* B. cysticola associated with epitheliocystis (Toenshoff et al. 2012; Mitchell et al. 2013). Simultaneous administration of oligo probes BraCy-129 with any of the HK genes; aceE/lepA/rplB/rpoC all showed identical specificity in spatial expression (Fig. 2A, B). The co-localization of mRNA targeted by these probes thus confirms that the newly acquired HK sequences originate from *Cand.* B. cysticola (Fig. 2C).

The observable differences in emission intensity from the separate probes also seems to correspond with the genetic expression levels revealed by RT-PCR. Overlay of the separate fluorophores (Fig. 2C) showed an increased level of emitted fluorescence from BraCy-129 compared to the probes of putative HK genes (Fig. 2C). As with the application of RT-PCR assays, this is to be expected due to the highly expressed nature and the putative multiple copies of the 16S rRNA gene being transcribed and subject to RNA hybridization. However, differences in quantum yield of these fluorophores or the specific sequences of these probes could also have contributed to the observed differences in intensity (Mujumdar et al. 1993; Kretschy et al. 2016).

## Conclusions

The specificity demonstrated by RT-PCR assays and concordance in spatial expression of HK gene FISH probes and the previously reported 16S probe for *Cand.* B. cysticola confirms that these newly acquired HK sequences originate from *Cand.* B. cysticola. This information constitutes a major prerequisite for future phylogenetic studies of this bacterium’s population structure.
